# Searching for Factors that Distinguish Disease-Prone and Disease-Resistant Prions via Sequence Analysis

**DOI:** 10.4137/bbi.s550

**Published:** 2008-03-12

**Authors:** Kanaka Durga Kedarisetti, Scott Dick, Lukasz Kurgan

**Affiliations:** Department of Electrical and Computer Engineering, University of Alberta, Edmonton, Alberta, Canada

**Keywords:** prions, prion misfolding, point mutations, sequence alignment, exchange groups, conformational transition

## Abstract

The exact mechanisms of prion misfolding and factors that predispose an individual to prion diseases are largely unknown. Our approach to identifying candidate factors in-silico relies on contrasting the C-terminal domain of PrP^C^ sequences from two groups of vertebrate species: those that have been found to suffer from prion diseases, and those that have not. We propose that any significant differences between the two groups are candidate factors that may predispose individuals to develop prion disease, which should be further analyzed by wet-lab investigations. Using an array of computational methods we identified possible point mutations that could predispose PrP^C^ to misfold into PrP^Sc^. Our results include confirmatory findings such as the V210I mutation, and new findings including P137M, G142D, G142N, D144P, K185T, V189I, H187Y and T191P mutations, which could impact structural stability. We also propose new hypotheses that give insights into the stability of helix-2 and -3. These include destabilizing effects of Histidine and T188-T193 segment in helix-2 in the disease-prone prions, and a stabilizing effect of Leucine on helix-3 in the disease-resistant prions.

## Introduction

Misfolding of the prion protein (PrP) is believed to be responsible for the Transmissible Spongiform Encephalopathy (TSE) diseases (Prusiner, 1998). Experimental investigations suggest that the pathogenesis of TSE is characterized by the unfolding of the normal Prion protein (PrP^C^) followed by misfolding into an infectious “scrapie” isoform (PrP^Sc^) (Pan et al. 1993). According to the protein-only hypothesis, PrP^Sc^ promotes structural conversion of the cellular PrP^C^ into the pathogenic conformation (Prusiner, 1998; Prusiner et al. 1998). The pathogenesis presumably involves the initial formation of PrP^Sc^, which is a result of a point mutation(s) or some exogenous factors, and which subsequently interacts with and converts PrP^C^ molecules into PrP^Sc^ molecules. The last decade of research has provided a significant amount of evidence that supports this hypothesis (Mead, 2006).

**Figure 1:**
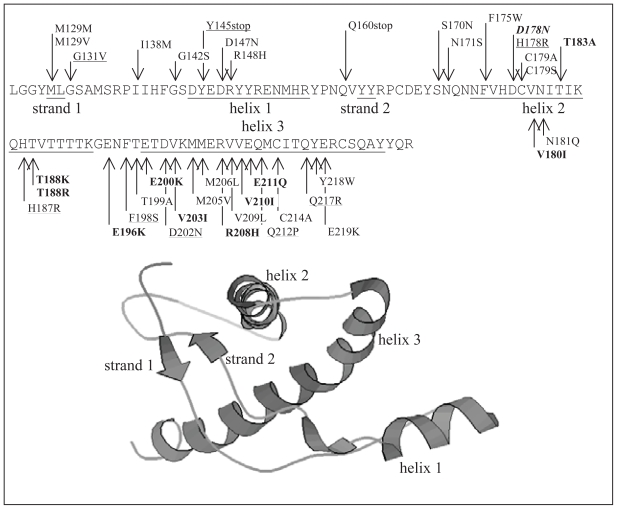
Sequence and mutations in the C-terminal domain of huPrP together with the ribbon drawing of the corresponding 3D structure (positions 125 to 228 of 1HJM). The secondary structure segments are denoted by underscores. **Bold** indicates pathogenic mutations associated with the CJD phenotype, underline indicates GSS, and *italic* indicates FFI.

Known PrP^C^ structures reveal that the C-terminal domain (positions 125 to 230) is structured and contains three α-helices and a short β-sheet that includes two strands (see Fig. 1), whereas the N-terminal domain (positions 23 to 126) is highly flexible and cannot be assigned a particular conformation (Riek et al. 1997; Riek et al. 1998; Lopez-Garcia et al. 2000). At the same time, the structure of the PrP^Sc^ isoform is currently still unknown.

Spectroscopic studies have shown that PrP^C^ is composed of about 42% α-helices and 3% β-sheets, whereas PrP^Sc^ is composed of only 30% α-helices and 43% β-sheets (Pan et al. 1993). Thus, the conformational transition of PrP^C^ into PrP^Sc^ has to involve unfolding of some α-helices and formation of new β-sheets. Helix-1 is the most conserved in PrP sequences and forms only a few interactions with the rest of the C-terminal domain. These facts led to a model in which helix-1 was considered as a starting point for conformational transition and forms a β-like aggregate, whereas helix-2 and helix-3 retain their conformation (Huang et al. 1995; Morrisey et al. 1999; Wille et al. 2002). Some recent models of the pathologically misfolded form of PrP also show that the helix-1 region is unstable and has to unfold during the conformational transition (Eghiaian et al. 2004). At the same time, recent results provide strong evidence that helix-1 is not converted into a β-sheet during the aggregation of PrP^C^ to PrP^Sc^ (Watzlawik et al. 2006). This conclusion is also supported by experimental data obtained using low-resolution electron crystallography which suggest that helix-1 in PrP^Sc^ refolds into a left-handed β-helix (Wille et al. 2002), while subsequent work shows that helix-1 is not included in the β-helix but forms an unstructured loop (Govaerts et al. 2004). These discrepancies motivate this work, in which we use sequence based analysis to find factors that could impact the stability of particular secondary structure segments.

A number of point mutations in the human prion have been identified. A significant proportion of all mutations are found within the structured C-terminal domain; 27 out of total of 30 as reported in (Kovacs et al. 2002) and 37 out of 55 as reported in PrionDB at http://www.receptors.org/Prion/ (Horn et al. 2001). Thus, we focus our attention on the C-terminal domain (see Fig. 1). Pathogenic mutations are classified based on their association with prion diseases that include Gerstmann-Straussler-Scheinker disease (GSS), Creutzfeld-Jakob disease (CJD), and Fatal Familial Insomnia (FFI). The number of possible single-point mutations in the C-terminal domain is relatively large (109 positions * 19 = 2071), and thus it is not feasible to physically check every one of them using wet-lab techniques. Well-designed computational experiments (such as the design we propose) can reveal promising candidate factors, which serve as new hypotheses for wet-lab investigation. To this end, another of our goals is to use sequence based analysis to find point mutations that could predispose PrP^C^ to misfold into PrP^Sc^.

In contrast to other sequence analysis based approaches that contrast prion proteins with structurally similar proteins such as Doppel (Kuznetsov and Rackovsky, 2004), we present a novel in-silico approach based on the assumption that some species are susceptible and others are resistant to prion disease (PD). We divide the available prion sequences from vertebrate animals into those that are prone to PD, and those that are apparently resistant, i.e. there are no reports of any known PD in that species and research suggests that they do not develop PD. We then compare the PrP sequences from these two groups (hereafter “the contrasts”), with a focus on the C-terminal domain. To the best of the authors’ knowledge, only two prior sequence-analysis-based contributions perform similar contrasting analysis, but they focused on identification of β-aggregating stretches (Tartagia et al. 2005) or contrasted just four prion proteins (Pappalardo et al. 2007). We used an array of computational techniques including multiple sequence alignment, exchange group similarities, and feature selection methods to identify possible factors that distinguish the contrasts for a larger set of 11 proteins. We suggest that such discriminating factors are potentially important in the conformational change from PrP^C^ to PrP^SC^. The results of this analysis are best viewed as either evidence confirming known factors associated with prion misfolding, or newly hypothesized factors that predispose the misfolding.

## Materials and Methods

### Dataset

We extracted the sequences of all prions that were deposited in Protein Data Bank (PDB) (Berman et al. 2000) as of September 2007. This database is expert-curated, which assures high quality of the data, and includes structural information, which allows us to identify secondary structure regions and perform structural analysis. The 70 prion sequences stored in PDB belong to 15 species: chicken (1 sequence), ovine (4 sequences), human (29), elk (1), rabbit (1), canine (1), frog (1), turtle (1), bovine (5), mouse (4), cat (1), pig (1), syrian hamster (2), sheep (5), and yeast (13). Yeast prions were removed since they have no homology with the remaining vertebrate prions, and are shown to have substantially different properties (Bousset and Melki, 2002). We filtered out redundant sequences, i.e. we selected the newest deposition for each species (except for sheep prions, for which there are two depositions from 2004; we selected the slightly older 1UW3 that does not include polymorphisms), and eliminated sequences that did not cover the C-terminal domain. We note that among the C-terminal domain sequences the four bovine sequences and the two mouse sequences are identical, while the only differences between the two sheep sequences are C148R and Q168H mutations, and among ten human prions nine sequences are identical and one differs from them by two mutations M166C and E221C. The positions associated with these mutations do not show any consistent pattern vs. our contrasts (i.e. they do not serve to differentiate PD-prone from PD-resistant species), and so the duplicate sequences are redundant and could be safely removed. It is in fact necessary to remove them; data-mining techniques such as feature selection assume that there is no redundancy in a dataset (deletion of redundant data items is a standard preprocessing step in data mining), and so the presence of redundant sequences would undermine our results.

Next, for the remaining 14 species we searched for evidence in the literature that supports existence of PD, or which suggests that they are PD resistant. Eight mammalian species (human, bovine, sheep, elk, cat, mouse, syrian hamster, and ovine) are shown to develop PD (Prusiner, 1997; Prusiner, 1998; Benkel et al. 2007; Murayama et al. 2007). At the same time, prion diseases were never confirmed for the non-mammalian species turtle, chicken and frog, and several studies suggest that they do not develop prion diseases (De Simone et al. 2006; Ji et al. 2007). For the remaining 3 species, i.e. pig, canine, and rabbit, we could not find sufficient evidence to categorize them to either class (Wells et al. 2003; Vorberg et al. 2003; Lysek et al. 2005). We note that canine shares high sequence similarity with PD-prone species, i.e. between 88% for human and 98% for cat, and moderate similarity with PD-resistant species, i.e. between 30% for frog and 41% for turtle. Similarly for rabbit and pig the sequence similarity to PD-prone species ranges between 90% (for human) and 96% (for sheep and ovine), and between 86% (for hamster) and 93% (for elk), respectively, while for PD-resistant the similarity ranges between 31% (for frog) and 42% (for turtle), and between 28% (for frog) and 41% (for turtle), respectively. It is of course possible to simply add the three uncategorized species to the “PD-resistant” class, since no evidence has been produced that they do experience prion disease. However, this would, in our view, be a serious methodological error. Our analysis contrasts species that are known to develop PDs against those that clearly do not, and this distinction directly affects all of the computational techniques (discussed below) that will be employed in our work. The inclusion of pig, canine and rabbit prions would undermine the contrasts, because we could not positively assert that these are truly PD-resistant species. Our methods are fundamentally intended to identify only those differences that perfectly distinguish between the two classes; if the classes themselves become uncertain, our entire methodology becomes merely a “shotgun correlation.” The eleven species we have selected already represent the maximal set of species that we can confidently differentiate into our two classes at the present time. It would be highly desirable to include more species in each class; data-mining techniques such as feature selection are generally intended to operate over thousands or tens of thousands of examples. Obtaining a firm determination of susceptibility to prion disease in canines, rabbits and pigs would be an excellent start.

### Point mutations

We performed multiple sequence alignment of the 11 PrP C-terminal domain sequences using ClustalW version 1.83 (Chenna et al. 2003). ClustalW produces biologically meaningful alignments that allow finding identities, similarities and differences between a set of protein sequences. Next, we searched for significant mutations based on positions that are conserved within PD-prone and PD-resistant species. Each position was categorized as follows:

1. Each position that includes a conserved (the same) amino acid (AA) in the PD-prone species and a conserved (the same) AA (different from the AA conserved for the PD-prone) in the PD-resistant species is categorized as significant. Such a position shows conservation within each group while at the same time it differentiates the contrasts.
2. Each position that has different AAs over different PD-prone species and/or PD-resistant species is categorized as insignificant. These positions show no significant conservation pattern.
3. Each position that has conserved (the same) AA over all PD-prone and resistant species is considered insignificant. Although these positions show significant conservation, these residues do not differentiate the contrasts.

Working from the hypothesis that TSE mutations are exclusive to PD-prone species, each significant position is a candidate factor that predisposes PrP^C^ to misfold into PrP^Sc^.

We repeated the same procedure using exchange groups, which represent conservative replacements of AAs through evolution (Dayhoff et al. 1978). They relax the constraint of conservation by defining equivalence classes of AAs, as derived from the BLOSUM AA substitution matrix (Henikoff and Henikoff, 1992), which in turn was derived based on the BLOCKS database (Henikoff and Henikoff, 1991). This reduces the alphabet of 20 AAs to the following six exchange groups: E_1_ = {H,R,K}, E_2_ = {D,E,N,Q}, E_3_ = {C}, E_4_ = {S,T,P,A,G}, E_5_ = {M,I,L,V}, and E_6_ = {F,Y,W}, and we consider a position to be conserved if all corresponding AAs belong to the same exchange group. We then label each position according to the three rules above, using exchange groups instead of individual AAs. Again, any position with conserved (but different) exchange groups in PD-prone and resistant species is another candidate factor that predisposes PrP^C^ to misfold into PrP^Sc^.

### Stability of secondary structure

Each prion sequence was converted into a feature-based vector, and the features that differentiate the contrasts were identified using a combination of feature selection methods and correlation analysis. The features represent physicochemical properties of protein sequences that were previously used to characterize and predict certain properties related to the secondary structure of protein sequences, including structural class (Feng et al. 2005; Cao et al. 2006; Kedarisaetti et al. 2006; Kurgan and Chen, 2007) and secondary structure content (Zhang et al. 2001; Ruan et al. 2005; Homaeian et al. 2007). As such, features that discriminate between the contrasts are candidate factors that predispose β-sheet poor PrP^C^ to misfold into β-sheet rich PrP^Sc^. As the conformational change from PrP^C^ to PrP^Sc^ will ultimately be driven by physiochemical properties, these features are a promising source of candidate factors. The features we analyze include:

- Molecular weight, *MolW* (Kedarisaetti et al. 2006; Homaeian et al. 2007), of a protein sequence is the result of adding up the average molecular weight *MolW_i_* values of its residues (see Table. 1) plus the mass of a water molecule (*MolW*_*H*_2__*_O_*) that is approximately 18 daltons
  $$MolW=MolW_{H_{2}O}+\sum_{i=1}^{N}MolW_{i}$$
  where *N* denotes the total number of residues in the sequence.
- Average isoelectric point, *pI* (Kedarisaetti et al. 2006; Kurgan and Chen, 2007; Homaeian et al. 2007), of a protein sequence is computed based on the average isoelectric point *pI_i_* values of its residues (see Table. 1)
  $$pI=\frac{1}{N}\sum_{i=1}^{N}{pI}_{i}$$
- Composition vector, *CV*, and composition moment vector, *CMV* (Zhang et al. 2001; Feng et al. 2005; Ruan et al. 2005; Cao et al. 2006; Kedarisaetti et al. 2006; Kurgan and Chen, 2007; Homaeian et al. 2007) are defined as the composition percentage of each residue in the sequence that incorporates information about the position of residues
  $$CMV_{i}^{k}=\frac{\sum_{j=1}^{n_{i}}{n^{k}}_{ij}}{\prod_{d=0}^{k}\left(N-d\right)}$$
  where *n_ij_* represents the *j*th position of the *i*th amino acid, *n_i_* is the frequency of *i*th amino acid in the sequence, and *k* is the order of the CMV. We apply CMVs for *k* = 0, 1, 2. Note that *CMV_i_^0^* reduces to *CV_i_*.
- Order *n* hydrophobicity auto-correlation function, *A_n_^a^* (Zhang et al. 2001; Kedarisaetti et al. 2006; Homaeian et al. 2007; Kurgan and Chen, 2007), is computed by summing up the products of amino acid indices *a_i_* (see Table 1) of every pair of residues separated by *n* residues.
  $$A_{n}^{a}=\frac{1}{N-n}\sum_{i=1}^{N-n}a_{i}a_{i+n}$$
  where *a* denotes the following hydrophobicity indices: Fauchere-Pliska’s (*FH*) index (Fauchere and Pliska, 1983) with *n* = 1,2, …,10 and Eisenberg’s (*EH*) index (Eisenberg et al. 1984) with *n* = 1,2, …,6.
- sum, *H_sum_^a^*, average, *H_avg_^a^*, and 3-point running average, *H_sum3_^a^*, of the above hydrophobicity indices, (Kedarisaetti et al. 2006; Homaeian et al. 2007; Kurgan and Chen, 2007)
  $$\begin{array}{l} H_{sum}^{a}=\sum_{i=1}^{N}a_{i},H_{avr}^{a}=\frac{\sum_{i=1}^{N}a_{i}}{N},\\ H_{sum3}^{a}=\sum_{i=1}^{N-3}\left(\sum_{j=i}^{i+3}a_{j}\right)/3 \end{array}$$
  where *a* = {FH, EH}.
- Composition of property groups, *PG_i_*, where *i* denotes a given property (Cao et al. 2006; Kedarisaetti et al. 2006; Homaeian et al. 2007; Kurgan and Chen 2007). AAs are clustered based on their properties (see Table 2) and composition is computed for each of the groups and subgroups. The hydrophobicity group includes hydrophilic and hydrophobic AAs. R group classification is based on molecular weight, hydropathy and isoelectric point. Exchange groups cluster AAs based on accepted point mutations to represent conservative replacements through revolution. Electronic group classification is based on the tendency of AAs to accept or donate electrons. Other groups are defined based on molecular weights, polarity, aromaticity and charge. Finally, chemical groups are based on the composition of chemical groups that constitute the side chains, see Table 1.

We employed three feature selection techniques to minimize bias in our results. These are the ReliefF (Robnik-Sikonja and Kononenko, 2003), information gain (Quinlan, 1993), and the χ^2^ statistics, taken between a given attribute and the binary class (PD-prone/PD-resistant). The ReliefF algorithm estimates the ability of features to separate classes. This algorithm examines nearest-neighbors of a feature vector that belong to the same or a different class as the vector under consideration. Features that categorize these nearest neighbors correctly receive a high score, and the process is repeated for each feature vector. The second selection technique is based on the concept of minimization of *information entropy*, while the chi-square statistic measures deviation from an assumed (normal) distribution of values for independent variables. All three feature selection algorithms are implemented in the WEKA data-mining software package (Witten and Frank, 2005). As a cross-check on the three selection algorithms, we also compute the bi-serial correlation between each feature and the binary class variable.

## Results and Discussion

### Point mutations

**Figure 2:**
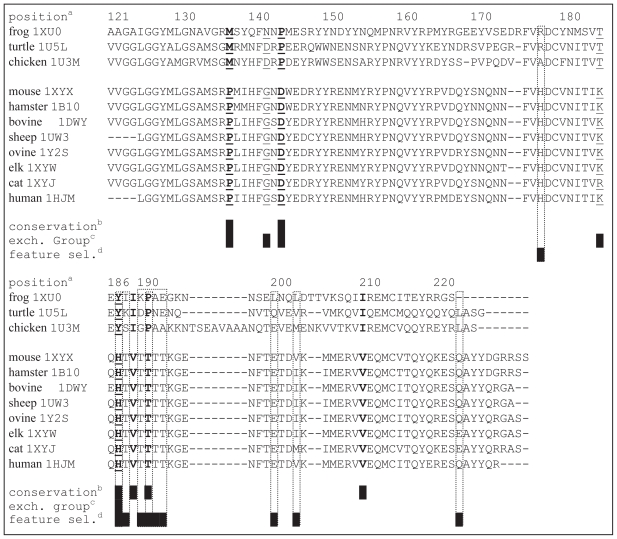
Results of sequence alignment between the three PD-resistant prions (top) and the eight PD-prone prions (identified by the PDB ID for the protein). ^a^Positions are encoded with respect to the huPrP. ^b^“Conservation” line shows positions (black squares with the corresponding residues shown in bold) that were significant based on the conservation of amino acids. ^c^“Exch. group” line shows positions (black squares with the corresponding residues underlined) that were significant based on the conservation of amino acids grouped in exchange groups. ^d^“Feature sel.” line shows positions (black squares with the corresponding residues denoted by dotted line boxes) that were significant based on the feature selection.

The aligned prion sequences are shown in Figure 2. Our analysis shows the following significant positions: 137, 144, 187, 189, 191, and 210, which are associated with the following point mutations with respect to huPrP: P137M, D144P, H187Y, V189I, T191P, V210I (see Fig. 2). Similarly, when considering conservation at the level of exchange groups, the following positions were found significant: 137, 142, 144, 185, and 187. The positions 137, 144, and 187 overlap with the results of residue conservation, while the remaining two positions are associated with G142D, G142N, and K185T point mutations. One mutation is a confirmatory result, while the remaining eight are new findings:

- P137M (new finding). Residues that compose helix-1 are not involved in hydrogen bonds with the rest of the C-terminal domain. This is true except for Y149 and Y150 which belong to helix- 1 and whose side chain hydroxyls donate to the carboxyl groups of D202 and the CO of P137 (Riek et al. 1998). Therefore, a mutation at P137 could further weaken interaction between helix-1 and the rest of the C-terminal domain. At the same time, several studies report importance of weakened interactions between helix-1 and other segments in the C-terminal domain on the folding into a stable native structure (Hirschberger et al. 2006; Schwarzinger et al. 2006; Eghiaian et al. 2007)
- G142D and G142N (new findings). A mutation at the same position, i.e. G142S, was previously classified as having a CJD-like phenotype (Gambetti et al. 2003). For this mutation, Glycine at position 142 was substituted with a polar, hydrophilic Serine. Using our approach, we identified mutations at that position involving Aspartate and Asparagine, which are very similar to each other and both also polar and hydrophilic, similar to the known mutation.
- D144P (new finding). Previous research shows that D144 forms a salt bridge with H140, R148 and R208 (Zuegg and Gready, 1999). The salt bridge between D144 and R208 links helix-1 and helix-3, while the R208H mutation is associated with CJD (Riek et al. 1998). Since salt bridges are suggested to increase the stability of proteins, mutation at this position could potentially lead to destabilization of the prion’s structure. Recent results also show that a point mutation leading to the disruption of a single salt bridge in p53 increases propensity to form amyloid fibrils (Galea et al. 2005).
- H187Y (new finding). This position is associated with a known H187R mutation that results in GSS (Cervenakova et al. 1999). At the same time, both Tyrosine and Arginine are polar and similar in size, i.e. their van der Waals volumes are 141 and 148, respectively.
- V210I (confirmatory finding). This mutation is well-known and is associated with CJD in humans (Riek et al. 1998).

We have shown that several of the new mutations we have found are closely related to known mutations involved in TSE diseases, while others may impact structural stability of the prion protein. While we were unable to find established research that would directly corroborate the remaining new mutations (K185T, V189I, and T191P), existing research indicates that mutations in this segment (which contains helix-2) may have β-sheet promoting effects. Helix-2 is characterized by a strong propensity for the extended conformation, and a single AA replacement in the vicinity of this helix is shown to significantly affect the conformational preference of the entire helix-2–helix-3 segment and to further increase the propensity for the extended conformation, facilitating conformational rearrangement in this region (Knaus et al. 2001; Kuznetsov and Rackovsky, 2004). These findings also correlate well with the high number of disease-promoting mutations in helices-2 and -3, which also points to the particular importance of these helices for conformational transition (only one disease-promoting mutation is found in helix-1 while seven and eight such mutations are found in helix-2 and helix-3, respectively).

### Stability of secondary structures

Our feature selection was performed using tenfold cross-validation to assure statistical validity for our results. Features are evaluated in each fold, and then they are ranked on their performance across all ten folds. Higher-ranked features have greater discriminatory power for the contrasts than lower-ranked ones. We average the ranks reported for each feature across our three feature selection methods. We report the top five features, ordered by average rank, which have biserial correlation coefficient values >0.9 in Table 3. The biserial correlation coefficient measures correlations between ratio-scale and binary variables, and is interpreted in the usual manner (values >0.8 indicate strong correlations).

**Figure 3:**
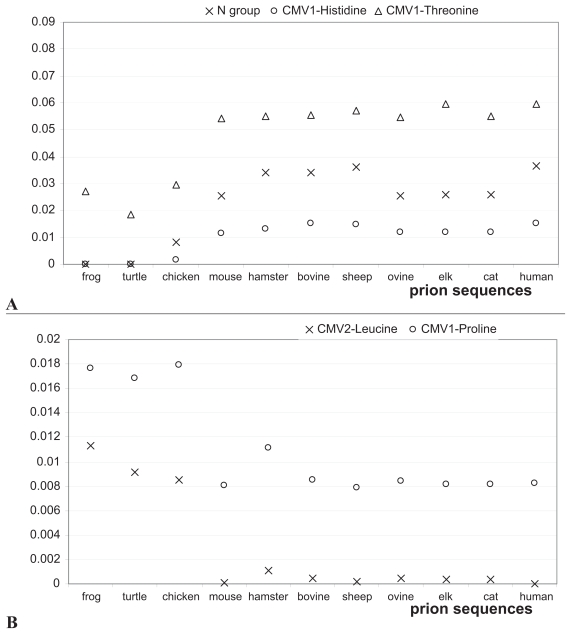
Values of top five features for the 11 prion sequences: features that indicate abundance of the associated amino acids in **A**) PD-prone prions, and **B**) PD-resistant prions. The first three sequences correspond to the PD-resistant prions, and the remaining sequences to the PD-prone prions.

The five features in Table 3 fall into two groups: those that show higher values for PD-prone species than PD-resistant species, and those that show higher values for PD-resistant species than PD-prone species. We begin our discussion with the former group. The second feature in Table 3 is related to the composition of the N group in the AA side chains. Since N group occurs only in Histidine, this feature indicates that presence of this AA is specific to one group of prion proteins. This finding is also supported by the third feature, *CMV_H_^1^*, which reveals additional details. Values of these two correlated features for the ten prion sequences are shown in Figure 3(a). The plots shows higher values of the composition moment vector for Histidine for the PD-prone species when compared with the PD-resistant species. Since the composition moment values are proportional to the distance of the corresponding residue from the N-terminal, high values indicate the presence of Histidine near the C-terminal in the PD-prone prions. Figure 2 shows two highly conserved Histidine positions in helix-2, i.e. 177 and 187, that are specific to PD-prone prions, while the only position in the PD-resistant chicken prion that contains Histidine is 140. This finding is supported by prior research which shows that charged Histidine side chains in the middle of α-helices have a destabilizing effect on the structure because of the unfavorable interaction with the helix macrodipole (Armstrong and Baldwin, 1993). This destabilizing effect in the context of protonation of H187 (Langella et al. 2004) provides some explanation for the weak stability of helix-2. We note that this finding can also be related to the H187R mutation, associated with GSS.

The *CMV_T_^1^* feature, which again is characterized by higher values for PD-prone species (see Fig. 3A) reveals that Threonine is significantly more abundant in this group of species. Figure 2 reveals that a highly conserved TVTTTT segment in helix-2 is specific to these prions. This segment is surface exposed and located between two glycosylation sites and most likely “covered” by the glycan side chains. It was previously found to be significant in the context of a potential molecular mechanism leading to the destabilization of the helix-2 segment, which postulates formation of a hydrogen bond between residues T188 and T193 that drives the unwinding of the α-helix (Pappalardoa et al. 2004). Another study that looked into the TTTT sub-segment (positions 190–193) concluded that this sub-segment is usually found in a strand and/or loop conformation and that the second half of helix-2 would be better accommodated in non-helical conformations (Dima and Thirumalai, 2004).

In contrast, the remaining two features have higher values for the PD-resistant prions; see Figure 3B. Analysis of the aligned sequences shown in Figure 2 reveals that although Leucine is present at positions 125, 130, and 138 in both types of prions, this AA is only present in the vicinity of the C-terminal in the PD-resistant prions. As a result, positions 200, 203, and 223 (located within helix-3) were identified as significant locations based on the position-sensitive *CMV_L_^2^* feature (see Fig. 2). Recent computational analysis of local interactions that promote formation of secondary structures shows that Alanine, Glutamine, Glutamate, and Leucine are strongly associated with formation of helices (Chen et al. 2006). We also note that positions 200 and 203 are associated with known mutations. Position 203 is associated with the V203I mutation that causes CJD (Peoc’h et al. 2000). E200K, which results in CJD, is one of the most common worldwide prion mutations (Mead, 2006). This mutation results in loss of a salt-bridge interaction between the side chains of E200 and K204 (Zhang et al. 2000). In the native huPrP these side chains are intimately juxtaposed (within 5 Å) and therefore they could be involved in a salt bridge. In the E200K mutant protein, the nearest negatively charged side chain to E200 is that of D196 which is 13 Å from E200 (Zhang et al. 2000). Therefore, mutation on this position could result in destabilization of the structure.

Finally, the *CMV_P_^1^* feature indicates that location 191, which contains a highly conserved Pro-line residue, is specific to the PD-resistant prions (see Fig. 2). We were unable to find existing research that would corroborate the significance of this position, due to the limited amount of work on non-mammalian prions.

## Conclusions

We present a novel, in-silico approach to identify factors related to misfolding of prion proteins. We contrasted PrP^C^ sequences of the C-terminal domains of PD-prone and PD-resistant species. The analysis focused on finding significant point mutations and investigating structural stability of secondary structures that comprise the C-terminal domain. We confirmed the V210I mutation, which is associated with CJD, and present several new findings that include P137M, G142D, G142N, D144P, K185T, V189I, H187Y and T191P mutations; destabilizing effects of Histidine and the T188-T193 segment on stability of helix-2 in the PD-prone prions; and stabilizing effects of Leucine on helix-3 in the PD-resistant species. All of these new findings are possible candidate factors that could influence conformational change from PrP^C^ to PrP^Sc^. They are a new set of hypotheses that should be investigated via wet-lab experimentation or (at a minimum) molecular dynamics simulations. In addition, if and when additional species can be definitively classified as PD-prone or PD-resistant, it would be quite interesting to repeat our experiments with these additional species included in the contrasts. Finally, we note that the resistance to prion diseases of the PD-resistant species could be a result of other factors besides the differences in their sequences, which should be addressed in future studies.

## Figures and Tables

**Table 1:** List of physicochemical amino acid indices and chemical groups used to derive features.

Amino acid	Code	Index	Physicochemical index/chemical groups				
			*MolW*	*pI*	*FH*	*EH*	Associated chemical groups
Alanine	A	1	71.0791	6.01	0.42	0.62	CH CO NH CH_3_
Cysteine	C	2	103.1437	5.07	1.34	0.29	CH CO NH CH_2_ SH
Aspartate	D	3	115.0887	2.77	−1.05	−0.9	CH CO NH CH_2_ CO COO^−^
Glutamate	E	4	129.1157	3.22	−0.87	−0.74	CH CO NH CH_2_ CH_2_ CO COO^−^
Phenylalanine	F	5	147.1772	5.48	2.44	1.19	CH CO NH CH_2_ CAROM CHAROM CHAROM CHAROM CHAROM CHAROM
Glycine	G	6	57.0521	5.97	0	0.48	CH_2_ CO NH
Histidine	H	7	137.1414	7.59	0.18	−0.4	CH CO NH CH_2_ CAROM CHAROM N CHAROM NH
Isoleucine	I	8	113.16	6.02	2.46	1.38	CH CO NH CH_2_ CH CH_3_ CH_3_
Lysine	K	9	128.1792	9.74	−1.35	−1.5	CH CO NH CH_2_ CH_2_ CH_2_ CH_2_ NH_3_^+^
Leucine	L	10	113.16	5.98	2.32	1.06	CH CO NH CH_2_ CH CH_3_ CH_3_
Methionine	M	11	131.1977	5.47	1.68	0.64	CH CO NH CH_2_ CH_2_ S CH_3_
Asparagine	N	12	114.104	5.41	−0.82	−0.78	CH CO NH CH_2_ CO C NH_2_
Proline	P	13	97.1171	6.48	0.98	0.12	CHRING CO NHRING CH_2_RING CH_2_RING CH_2_RING
Glutamine	Q	14	128.131	5.65	−0.3	−0.85	CH CO NH CH_2_ CH_2_ CO C NH_2_
Arginine	R	15	156.188	10.76	−1.37	−2.53	CH CO NH CH_2_ CH_2_ CH_2_ NH C NH_2_ NH_2_^+^
Serine	S	16	87.0784	5.68	−0.05	−0.18	CH CO NH CH_2_ OH
Threonine	T	17	101.1054	5.87	0.35	−0.05	CH CO NH CH CH_3_ OH
Valine	V	18	99.133	5.97	1.66	1.08	CH CO NH CH CH_3_ CH_3_
Tryptophan	W	19	186.2139	5.89	3.07	0.81	CH CO NH CH_2_ CAROM CAROM CAROM NH CHAROM CHAROM CHAROM CHAROM CHAROM
Tyrosine	Y	20	163.1756	5.67	1.31	0.26	CH CO NH CH_2_ CAROM CHAROM CHAROM CHAROM CHAROM CAROM OH

**Table 2:** Property groups of amino acids used to derive features.

Groups	Subgroups	AAs	Groups	Subgroups	AAs
R groups	Nonpolar aliphatic	AVLIMG	Hydrophobicity groups	Hydrophobic	VLIMAFPWYCG
	Polar uncharged	SPTCNQ		Hydrophilic basic	KHR
	Positively charged	KHR		Hydrophilic acidic	DE
	Negative	DE		Hydrophilic polar with uncharged side chain	STNQ
	Aromatic	FYW			
Exchange groups	E_1_	KHR	Electronic groups	Electron donor	DEPA
	E_2_	DENQ		Weak electron donor	VLI
	E_3_	C		Electron acceptor	KNR
	E_4_	AGPST		Weak electron acceptor	FYMTQ
	E_5_	ILMV		Neutral	GHWS
	E_6_	FYW		Special AA	C
Other groups	Charged	DEKHRVLI	Other groups	Tiny	AG
	Polar	DEKHRNTQSYW		Bulky	FHWYR
	Aromatic	FHWY		Polar-uncharged	NQ
	Small	AGST			

**Table 3:** Top five features that differentiate between PD-prone and resistant prions.

Feature	Avg. rank	Bi-serial correlation coefficient
*CMV_P_^1^*	7.6	0.97
Chemical N group	9.3	0.94
*CMV_H_^1^*	11.1	0.97
*CMV_T_^1^*	12.2	0.96
*CMV_L_^2^*	12.8	0.99
